# Food composition database development for between country comparisons

**DOI:** 10.1186/1475-2891-5-2

**Published:** 2006-01-19

**Authors:** Anwar T Merchant, Mahshid Dehghan

**Affiliations:** 1 Population Health Research Institute, McMaster University, Hamilton ON, Canada; 2 Department of Clinical Epidemiology and Biostatistics, McMaster University, Hamilton ON, Canada

## Abstract

Nutritional assessment by diet analysis is a two-stepped process consisting of evaluation of food consumption, and conversion of food into nutrient intake by using a food composition database, which lists the mean nutritional values for a given food portion. Most reports in the literature focus on minimizing errors in estimation of food consumption but the selection of a specific food composition table used in nutrient estimation is also a source of errors. We are conducting a large prospective study internationally and need to compare diet, assessed by food frequency questionnaires, in a comparable manner between different countries. We have prepared a multi-country food composition database for nutrient estimation in all the countries participating in our study. The nutrient database is primarily based on the USDA food composition database, modified appropriately with reference to local food composition tables, and supplemented with recipes of locally eaten mixed dishes. By doing so we have ensured that the units of measurement, method of selection of foods for testing, and assays used for nutrient estimation are consistent and as current as possible, and yet have taken into account some local variations. Using this common metric for nutrient assessment will reduce differential errors in nutrient estimation and improve the validity of between-country comparisons.

## Background

Nutritional assessment by diet analysis is a two-stepped process. The first step is the evaluation of food consumption, and the second the conversion of food into nutrient intake. To do this we need a food composition database, which lists the mean nutritional values for a given food portion. We then multiply food intake by the mean nutrient content of that amount of food (obtained from the food composition database) [1]. As most professionals conducting nutritional assessments are primarily concerned with the evaluation of food intake, a large part of the literature on nutritional assessment focuses on minimizing errors at this step. However, errors and discrepancies may arise in nutrient estimation from the food composition database, arising mainly from the assays used in nutrient estimation and the sampling procedure and date of the test foods [2]. Between-country comparisons are particularly prone to error when different food composition tables have been used to estimate nutrient intake. In recognition of these difficulties there are ongoing efforts since 1984 to standardize food composition databases over the world [2]. This is a continuous process because of increased global trade in foods, changes in fortification policies, development of newer assays for nutrient estimation, and addition of new foods in the global diet.

Over the last 40 years, global trade of foods grew in both developed and developing countries [3]. Import of foodstuffs in developing and developed counties grew by 115% and 45% respectively. Since the late 1990s, the least developed countries have become the major net importers of agriculture products [3]. Foods traded internationally include cereals, edible oils, animal products, sugar, fruits, vegetables, nuts, and coffee. Global food trade has also affected North American food consumption. Imports to America between 1980 and 1997 rose for fish from 45% to 62%, for fresh fruits from 24% to 34% and vegetables from 5% to 10% [3].

We have started recruiting participants into the Prospective Urban and Rural Epidemiologic Study (PURE), an investigation that evaluates the societal, familial, and personal determinants of diet and their relation with obesity and chronic disease globally. When recruitment is complete we anticipate participation from about 14 countries and approximately 120,000 persons. We are using the food frequency questionnaire (FFQ) to evaluate diet in this study as described earlier [4]. The use of a multi-country food composition database that would provide comparable data across these countries is a critical part of our nutrient assessment. **The objective of this paper is to describe our process and rationale for the selection and adaptation of food composition tables to make cross-country nutrient comparisons**.

### Review of food composition tables

#### The International Network of Food Data Systems (INFOODS)

The INFOODS is a global collaboration of persons with a stake in nutrition – scientists, health and agriculture professionals, policy makers, food industry personnel, and users [2]. It was formed in response to the need to establish uniform guidelines and methods for the nutritional assessment of foods so that the results would be comparable between countries. One of its main tasks is the organization and standardization of food composition databases the world over [2]. As a result of several years of work, the team developed and implemented a system of nomenclature and coding of foods and the development of regional databases [5]. In the revision of the food composition tables the investigators used standardized food codes and carefully scrutinized primary data sources for quality and reliability, types of assays used for nutrient estimation, units of reporting, and methods for dealing with missing data [6]. The documentation of the tables was also substantially improved. These steps substantially improved the quality and comparability of the food composition data but certain inconsistencies remained. The number of nutrients reported was not always constant across countries in the region [6]. The assays to determine nutrient content sometimes differed by country. It is also not clear how frequently these tables are updated.

#### The U.S. Department of Agriculture, Agricultural Research Service National Nutrient Database (USDA)

The U.S. Department of Agriculture, Agricultural Research Service, (USDA) National Nutrient Database for Standard Reference has information on over 7100 foods and up to136 components of foods, and is freely accessible via the Internet [7]. There is also an ongoing program of research and food testing to keep this database current. As the cost to estimate all the listed nutrients in one food is about US$12,000 [8], a selective approach to food testing is taken. Foods are chosen for analysis using the Key Foods approach [8]. Other factors such as the addition of newer foods in the diet [9], scientific interest in particular nutrients, or newer assays also guide the addition of nutrients and analysis of foods. For instance, in Release 12 selenium was added, and folate values updated following changes in fortification practices [10]. Even with these contingencies it is probably the most comprehensive, and regularly updated database of its kind available. For this reason it is used for nutrient estimation worldwide, often without consideration of differences. Moreover as fruits and vegetables are increasingly being imported into the US, their nutrient content in all likelihood approach global averages.

## Methods

### Food composition database compilation

We used the USDA nutrient database as the primary nutrient data source for the PURE study because it is regularly updated, comprehensive and, the data are freely available. To ensure that the nutrient content of the foods were appropriate for the local countries, we referred to other sources such as the INFOODS food composition tables, or local food composition tables. As there are many entries for a single food (18 types of rice for instance) we developed the following algorithm to select the food from the USDA nutrient database that most closely matched the local food. To match the foods we considered total energy content and the following nutrients (macronutrients and minerals) for fruits and vegetables: energy, carbohydrates, calcium, phosphorous, sodium and potassium; dairy: energy, protein, fat, calcium, phosphorous; cereals: energy, carbohydrates, calcium, and phosphorous; and meats and eggs: energy, protein, fat, and iron, because these nutrients were likely to be present in those food groups. We only used macronutrients and minerals for matching because the assays for these nutrients have high within laboratory agreements [11]. We did not include vitamins in the matching process because their estimation is sensitive to the assay, method of food preparation, and storage [12-15].

**Figure 1:**
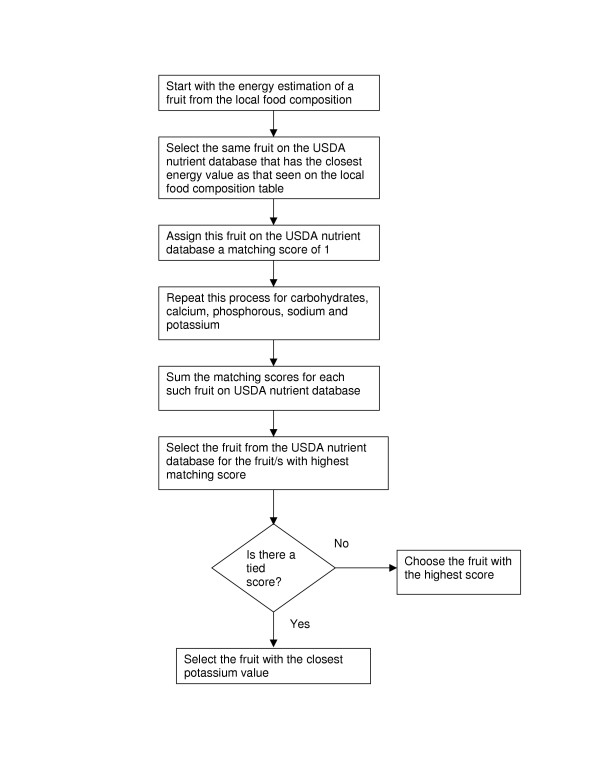
Algorithm to select a fruit from the USDA nutrient database using local food composition database as a starting point.

To select the food most similar to the local food we started with the estimated nutrient intake from the local food composition table. We first compared total energy intake for 100 g of that food estimated from the local food composition table. The food with the most similar total energy intake per 100 g of that food on the USDA nutrient database was given a matching score of 1. We repeated this process with the next nutrient and so on, until all the entries for that particular food group were exhausted. The food in the USDA nutrient database with the highest total watching score was considered as being the closest to the local food. In case of a tie we considered the closest match with potassium for fruits and vegetables, total fat for dairy and meats, protein for eggs, and carbohydrates for cereals. The algorithm to select fruits is described in Figure 1. We also considered the following additional rule. For fruits and vegetables we considered raw or unprocessed categories only unless specified otherwise. The process to select rice using this algorithm is described in Table 2. As the methods for rice preparation differ from place to place, we selected rice from the USDA nutrient database using the nutrient values of uncooked rice and then calculated the nutrient value for cooked rice for each country.

**Table 1:** Status of local food composition tables in some PURE participating countries

**Countries**	**First publication**	Date of update*	**Maximum No. of food items**	**Maximum No. of nutrients**
Argentina	1935 – 1942	1992	280	16
Brazil	1950	2002	1062	33
Colombia	1944	1990	600	32
Chile	1961	1997	≅2000	25
Zimbabwe	1989	Not available	201	18
UAE	Do not exist	Do not exist	Do not exist	Do not exist
USDA, SR18	1973	2004	7146	136

* Note: The date of update is not the date of the assay.

**Table 2:** Selection of rice from USDA nutrient database for specific countries using the algorithm described

**Country specific**	**Food Description**	**Water/g**	**Energy/kcal**	**Protein/g**	**Lipid/g**	**CHO/g**	**Ca/mg**	**P/mg**	**K/mg**	**Na/mg**
Argentina	Arroz, grano, blanco, pulido, crudo	12.5	346	6.9	0.2	79.2	9	93	78	4
Brazil	Arroz branco, curdo		365	7.14	0.66	80	28	115	115	5
Colombia	RICE, WHOLE, RAW	12.9	360	6.6	0.6	79.3	9	108	86	1
Chile	Arroz	12	365	7.1	0.7	79.5	28	115	115	5
Zim_FCT	Rice	122	357.4	6.8	0.6	80.6	8.6	109.5	95.3	14.3
**USDA NDB_No**										
20040	RICE, BROWN, MEDIUM-GRAIN, RAW	12.37	362	7.5	2.68	76.17	33	264	268	4
20044	RICE, WHITE, LONG-GRAIN, REG, RAW, ENR	11.62	365	7.13	0.66	79.95	28	115	115	5
20046	RICE, WHITE, LONG-GRAIN, PARBLD, ENR, DRY	9.7	374	8.11	1.04	80.43	55	156	187	3
20048	RICE, WHITE, LONG-GRAIN, PRECKD OR INST, ENR, DRY	8.38	380	7.82	0.94	82.32	22	118	27	10
20049	RICE, WHITE, LONG-GRAIN, PRECKD OR INST, ENR, PREP	72	117	2.18	0.5	25.1	8	37	9	4
20050	RICE, WHITE, MEDIUM-GRAIN, RAW, ENR	12.89	360	6.61	0.58	79.34	9	108	86	1
20052	RICE, WHITE, SHORT-GRAIN, RAW	13.29	358	6.5	0.52	79.15	3	95	76	1
20054	RICE, WHITE, GLUTINOUS, RAW	10.46	370	6.81	0.55	81.68	11	71	77	7
20056	RICE, WHITE, WITH PASTA, DRY	7.13	368	9.37	2.44	75.32	46	158	209	1866
20060	RICE BRAN, CRUDE	6.13	316	13.35	20.85	49.69	57	1677	1485	5
20061	RICE FLOUR, WHITE	11.89	366	5.95	1.42	80.13	10	98	76	0

NDB_No 20044 chosen for Brazil, Colombia, Chile

NDB_No. 20050 chosen for Zimbabwe and UAE

NBD_No 20052 chosen for Argentina

For countries for which there was no existing food composition table, UAE and Kuwait for instance, we included all the data from the USDA nutrient database. In this case we chose a generic food variety. For instance, for orange we chose "oranges, raw – all commercial varieties (NDB No: 09200)". Similarly for apples we used the mean nutrient content of a generic apple with and without the skin, after determining from local nutritionists how the apple is eaten in those countries.

For fruits and vegetables that were not available in USDA; we took two approaches. First, we found the common name of the local food item and matched it with the same scientific name in English, and then chose a similar food item from USDA. For example, taro a vegetable consumed in Zimbabwe and its name is *Colacasia antiquorum *and is from the yam family. Therefore, we used yam nutrient content for taro.

Second, if the scientific name was not available, we searched for a scientific description of the food from horticulture sources. For example as gourds are very closely related to cucumbers [16], we used the nutrient content of cucumber with peel (USDA code 11494) from USDA for gourds. For foods not found in the USDA nutrient database but available in the local food composition tables (caterpillars in the case of Zimbabwe for example), we used other sources (Korean sushi caterpillar from the ESHA database for instance) [17].

We called the food composition table constructed in this way the PURE-USDA food composition table. Such a table has all the 137 nutrients found in the USDA SR18 nutritional database, and the number of food items that are found in the respective country specific food frequency questionnaire.

### Estimation of nutrient content of mixed dishes

To estimate the nutrient content of mixed dishes we asked nutritionists in each of the participating countries to send us typical recipes that were commonly eaten in their countries. Recipes contained the weight in grams of raw ingredients. For each recipe, we calculated the nutrient content of the dry matter of each constituent. We then applied the retention factors for the nutrients based on the cooking method, using data from the Agriculture Handbook No. 102 [18] Cooking at a high temperature, for instance, destroys folate. The yield of recipe is considerably different from the dry weight of the ingredients depending on the cooking method. Beans absorb water when cooked, for instance, and a cup of dried beans can become 2–3 cups of cooked beans. Based on the cooking method we then applied yield factors, from the USDA handbook, to adjust for changes in total weight due to preparation and cooking USDA[9]. We then divided the total yield of the mixed dish from the recipe by the number of servings the recipe made and calculated the weight of the serving, energy and nutrient content for one serving and 100 g of the mixed dish. In the food composition table we entered the nutrient content of 100 g of the mixed dish.

For example, 100 g of uncooked beans contain 89 g dry matter. Suppose 3 cups of uncooked beans (582 g) become 6 servings when cooked, the dry matter in the 3 cups of beans will be 582 g * 89/100 = 518 g. The retention factor for calcium, however, is 0.85, which means that 85% of the calcium found in uncooked beans is retained after cooking. To estimate the yield we referred to the USDA handbook of yield factors, which for beans is 1.52. This means that 100 g of dry beans become 152 g of beans when cooked; 3 cups of dried beans would therefore become (582 g × 1.52 = 884.6 g) of cooked beans. As this was equivalent to 6 servings each serving size was 147 g. We made similar calculations for all the other nutrients that were evaluated.

### Data collection for typical recipes

To demonstrate the impact of using different food composition databases in nutrient estimation of recipes we used recipe data from Argentina, Brazil, UAE and Zimbabwe. Local nutritionists sent us typical recipes of beef or bean stew that were commonly eaten in these countries. We analyzed the recipes as described above and estimated the nutrient content of each of the four recipes using three different nutrient databases: local nutrient database, PURE-USDA nutrient database for that country, PURE-USDA nutrient database for Zimbabwe.

To assess the impact on nutrient estimation from FFQ data using different food composition databases we selected people who reported eating stew at least once a month on FFQ administered in (Argentina, N = 57, Brazil, N = 100, UAE, N = 99). For each of these people we calculated nutrient intakes from stew using two databases: PURE-USDA nutrient database specific to their particular country and PURE-USDA nutrient database for Zimbabwe and calculated intra class correlation coefficients between selected nutrients.

## Results

The last time the local food composition databases were partially updated ranged from 3–15 years ago, as compared with one year for USDA's SR18 (Table 1). The number of food items ranged from 201–2000, and maximum number of nutrients from 16–33 for the local food composition databases as compared with 7,146 food items and 136 nutrients for USDA's SR18. The local food composition databases did not provide detailed information about how the test foods were sampled, or the assays that were used for nutrient estimation, as compared with detailed documentation that was available for the USDA nutrient database.

Table 2 contains a list of the different types of uncooked rice in the USDA nutrient database, and the nutrient values for uncooked rice from the respective local food composition tables. At the end of the table there is the result of applying our proposed algorithm to select the rice in the USDA nutrient database that is closest in nutrient content to the local rice.

The nutrient content of four recipes of stew (100 g) varied depending upon the food composition table that was used in the estimation in all the countries that we examined (Table 3). For instance, carbohydrate intake estimated from the PURE-USDA database for Argentina was 11.0 g versus 12.5 g estimated using the same data but the PURE-USDA database for Zimbabwe (Table 3). There were differences in micronutrient and macronutrient estimations depending upon the choice of the database.

**Table 3:** Nutrients content of 100 g beef stew analyzed against three food composition tables: Local, country specific PURE-USDA, and Zimbabwe-USDA food composition tables

**Country**	**Energy/kcal**	**Protein/g**	**Fat/g**	**CHO/g**	**Ca/mg**	**P/mg**	**Fe/mg**	**K/mg**	**Na/mg**	**VitA/Req**	**VitC/mg**	**Folate/ mg**
**Argentina**												
Local	131	10	2.3	12.6	26.6	50.6	0.8	346	366	NA	16.1	NA
PURE-USDA	118	5.6	5.9	11	16.5	83	0.7	357	364	49.6	16.9	13.8
Zimbabwe-USDA	114	7.2	4.0	12.5	14.4	71.6	1.2	346	367	46	13.1	5.0
**Brazil**												
Local	371	7.2	29	20.4	27.7	67.4	1.9	18.4*	305.3	9.7	1.5	1.2
PURE-USDA	303	7.7	22	19.2	36.2	95.1	1.3	309	443.6	0.03	1.3	33.5
Zimbabwe-USDA	300	1.4	21.8	19.3	21.8	101.5	1.2	264.9	443.4	0.03	0.7	46.3
**Zimbabwe**												
Local	225	12.7	18.4	2	12.6	78.2	1.8	187	1007	37.7	4.9	9.5
PURE-USDA	161.3	18.3	8.4	2.	11.2	109	2.1	201.6	1007	4.6	2.45	7.7
UAE												
USDA	167.5	11.8	10.7	5.6	12.7	75.6	1.4	210	221.2	2.2	6.17	7.5
Zimbabwe-USDA	106.3	11.7	4.02	5.4	11.2	79	1.5	219	200.3	2.8	5.05	6.5

* Potassium (K) for beans is not available on Brazil FCT this might be the major source of underestimation

The intraclass correlations using FFQ data comparing nutrient intake from stew estimated using two nutrient databases both derived from the USDA nutrient database were generally good, but sometimes varied for macronutrients and micronutrients (Table 4). For instance the intraclass correlation coefficient for folate in Argentina was 0.78, for protein in Brazil it was 0.54, and for fat in UAE it was 0.79 (Table 3).

**Table 4:** Comparison of nutrient estimates of 100 g beef stew analyzed by Pure-USDA and Zimbabwe-USDA nutrient databases using mean intake and intraclass correlation coefficients (ICC)

**Country**	**Energy/kcal**	**Protein/g**	**Fat/g**	**CHO/g**	**Ca/mg**	**P/mg**	**Fe/mg**	**K/mg**	**Na/mg**	**VitA/Req**	**VitC/mg**	**Folate/mg**
**Argentina**												
PURE-USDA	48.5	2.3	2.4	4.5	6.8	34.1	0.28	146.7	149.6	20.4	6.9	5.7
Zimbabwe-USDA	46.8	2.95	1.6	5.1	5.9	29.4	0.49	142.1	150.8	18.9	5.4	2.05
**ICC**	0.99	0.98	0.96	0.99	0.99	0.99	0.93	0.99	1	0.99	0.98	**0.78**
**Brazil**												
PURE-USDA	163.2	3.9	11.8	10.3	19.5	51.2	0.7	166.4	238.9	0.016	0.7	18.04
Zimbabwe-USDA	161.6	0.75	11.7	10.4	11.7	54.7	0.6	142.7	142.7	0.016	0.9	24.93
**ICC**	1	**0.54**	1	1	0.93	0.99	0.99	0.99	0.93	1	0.98	0.97
**UAE**												
USDA	31.7	2.24	2.03	1.06	2.4	14.3	0.26	39.8	41.9	0.42	1.2	1.4
Zimbabwe-USDA	20.1	2.21	0.76	1.02	2.1	15.0	0.28	41.5	38.0	0.53	0.95	1.2
**ICC**	0.95	1	**0.79**	0.99	0.99	0.99	0.99	0.99	0.99	0.98	0.99	0.99

(Argentina N = 57, Brazil N = 100, UAE N = 99)

## Discussion

Our strategy to improve between-country comparisons is to use a common food composition database from which to derive nutrient estimates of foods for all countries. The need to have a common food composition database was felt in an analysis of food composition databases of countries participating in the European Prospective Investigation of Cancer and Nutrition (EPIC) study [19]. Even though the nutrient estimates obtained for most nutrients were comparable some were not [20,21]. Nutrient estimates from FFQ data from Chile using American and British food composition tables had good overall agreement; the agreement was excellent for macronutrients (ICC for energy = 0.98, protein 0.98, carbohydrate 0.97), but less for few micronutrients (ICC = Iron 0.86, Zinc 0.91) [22].

When we compared two food composition databases, both derived from the USDA nutrient database, we found good overall agreement, but there were several notable exceptions (Tables 3, 4). This difference was there even though both tables were derived from the same version of the USDA nutrient database but only differed in the food that was selected. These differences in estimates therefore do not reflect the assay or method of test food selection, but likely true variation in nutrient content of the foods, or the different methods of cooking in the case of mixed dishes. This underscores the need to select the appropriate food from the USDA nutrient database. We did this by choosing the food in the USDA nutrient database that was most similar to the local food with respect to nutrients of interest. Moreover, the selection algorithm is reproducible, and we consistently applied it to develop the food composition databases for all the countries we studied. Any residual errors in nutrient content of foods resulting from this process were probably similar in all the country databases.

The errors that arise in nutrient estimation as a result of nutrient databases are systematic [23]. For instance, if an analytical method that underestimated fiber in foods was used in a food composition database, nutrient estimation using that nutrient database will consistently underestimate fiber. If different food composition databases were used to estimate nutrients in different countries the systematic errors would be different, and in all likelihood vary in magnitude and direction for different nutrients. Using different food composition databases for different countries would increase errors.

Even if food composition tables are based on sound principles, there are differences in estimates of nutrients depending upon which one is used for nutrient estimation. The advantage of using a common food composition database is that the errors are consistent between the countries, hence making data more comparable. For instance, if fiber intake is underestimated because of the method used to determine fiber content in the food composition database, it is equally underestimated in all the countries. This would impact estimates of absolute intake but probably not the relative ranking by fiber intake.

With increasing globalization the food available in different countries is not all grown in the region [3] and this is a potential source of errors in nutrient estimation. For instance, using the composition of an orange grown in Argentina for nutrient estimation would be erroneous if it were imported from elsewhere. To address this issue we consulted regional food composition databases and obtained the nutrient composition of a food commonly used in that country and matched it up with the food with the closest nutrient composition on the USDA list. Shai et al used a similar approach to develop a food composition database in Israel [24]. Choosing the food from the food USDA nutrient database that most closely resembles local foods would further reduce errors in nutrient intake estimation between countries. Another reason why our approach is less likely to be prone to error is the consistent method we used in estimating the nutrient content of mixed dishes. We measured dry matter and took into account retention and loss of nutrients as a result of food preparation. Many food composition tables did not consider yield and retention factors. Finally, local food composition tables do not have a comprehensive list of foods or nutrients; when these are missing the nutrient value from the missing food or nutrient are frequently taken to be zero resulting in error, when in fact they are missing.

Our approach also has some limitations. First, there are a fixed number of foods for which we have comparable food data. We therefore cannot use the common food composition database in its present form for the analysis of 24-hour recalls or food diaries but we will expand this list when we do study to validate our FFQs. However, all the foods on our FFQs are found in the table and it thus can be used to estimate nutrients from data generated with those FFQs and the tables can be updated to accommodate more foods. Second, the use of average portion sizes and categories of intake to estimate intake frequency are potential sources of errors in estimation using the FFQ, but these have been shown to be small [22]. Third, most importantly the errors are consistent [23], and thus not likely to influence the relative ranking of the persons by nutrient intake, and increasing the between country comparisons. Fourth, the USDA estimates may not be completely accurate [25], or the test foods in the USDA nutrient database may be dissimilar from the local foods [25]. Fifth, we cannot validate our method. To do that we would need to have a gold standard. In this case the gold standard would be nutrient estimates from country specific food composition tables, which would be prohibitively expensive to develop. However, the method has face validity, is reproducible, and removes potential errors arising from different assays to estimate nutrient content and food selection. Last, it is still possible that there may be large discrepancies in nutrient content of foods between the USDA database and what is found in the field because of manufacturing practices. For instance, an analysis of dietary sources of alpha-linolenic acid (from solid cooking oil) in Iran revealed very high trans fat contents [26], not accounted for by the USDA database. To correct for such anomalies it is necessary to periodically sample and chemically analyze selected commonly consumed foods.

We have prepared a food composition database primarily based on the USDA food composition database, from which to estimate nutrients using FFQ data from different countries. By using this method we have ensured that the units of measurement, sampling scheme for foods, and assays used for nutrient estimation are consistent and current, and have taken into account local conditions. Using this common metric for nutrient assessment will likely reduce differential errors arising from nutrient estimation and make the between country comparisons more valid.
